# The Association of Novel Single-Nucleotide Variants in the Collagen Matrix-Encoding Gene *PRDM5* with Aortic Aneurysmal Disease

**DOI:** 10.3390/life13081649

**Published:** 2023-07-28

**Authors:** Peyton Moore, Adam Wolf, Mohanakrishnan Sathyamoorthy

**Affiliations:** 1 Sathyamoorthy Laboratory, Department of Medicine, Anne Burnett Marion School of Medicine at TCU, Fort Worth, TX 76123, USA; p.moore@tcu.edu (P.M.); a.wolf@tcu.edu (A.W.); 2 Consultants in Cardiovascular Medicine and Science—Fort Worth (CCMS-FW), 1121 5th Avenue, Suite 100, Fort Worth, TX 76104, USA

**Keywords:** *PRDM5*, aortic aneurysm, aortic dissection, genetic basis of aortic disease, collagen matrix proteins

## Abstract

Thoracic aortic aneurysms are clinical conditions that are associated with severe clinical endpoints including dissection and rupture, potentially leading to sudden death. Contrary to their abdominal counterparts, thoracic aortic aneurysms are well-recognized to have a genetic basis underlying their development. Among all patients with aneurysmal disease who underwent clinical genetic screening in our program (N = 145), two patients were found to have variants of uncertain significance (VUS) in the *PRDM5* gene. This gene is responsible for multiple regulatory functions in extracellular matrix development, and this is the first report, to our knowledge, to associate this gene with aortopathy.

## 1. Introduction

Thoracic aortic aneurysms (TAAs) are uncommon clinical findings in cardiovascular medicine that may be defined as a dilation of all three layers of the aortic arterial wall with at least a 50% increase in diameter when compared to the contiguous aorta. The current estimated prevalence is 6 persons per 100,000 each year. Symptoms of TAA vary, with many being clinically silent prior to their eventual discovery on routine imaging [1]. Aneurysm formation is gradual and painless, which results in 95% of all cases remaining undetected until an acute aortic event such as dissection or rupture occurs, or until the expansion results in the compression of nearby structures [2]. The current understanding of TAA pathogenesis is limited, and the search for the etiology behind TAA development is heavily dependent on genetic testing. Genetic influence is evident in TAA expression, with as many as 20% of affected individuals having a first-degree relative with a dilated thoracic aorta [3]. The life-threatening complications of TAAs justify the importance of identifying genetic influences potentially responsible for aneurysmal development.

Suspected familial TAAs can be described as syndromic (20% of cases) or non-syndromic (80%). Syndromic classification includes aneurysm secondary to Marfan syndrome (MFS), Ehlers-Danlos syndrome (EDS), or Loeys-Dietz syndrome (LDS), while non-syndromic is further subdivided into sporadic and familial causes [2]. Syndromic causes of TAA result in cystic medial degeneration, a process that involves the degeneration of the elastic medial layer of the aorta. This process results in a loss of wall integrity and subsequent aneurysm formation [4]. Currently, 11 genes have sufficient clinical and scientific data to establish a highly penetrant risk for TAA and acute aortic dissections in patients with or without syndromic features. These 11 genes encode proteins involved in vascular smooth muscle cell contraction, extracellular matrix adhesion, transforming growth factor β signaling, or smooth muscle cell metabolism. These include the *FBN1*, *LOX*, *MYH11*, *ACTA2*, *MYLK*, *PRKG1*, *COL3A1*, *TGFBR2*, *TGFBR1*, *TGF-β2*, and *SMAD3* genes. Disease in families with an underlying genetic mutation in the absence of a genetic syndrome is typically inherited in an autosomal dominant fashion with decreased penetrance [5].

## 2. Case Descriptions

### 2.1. Case 1

**Figure 1:**
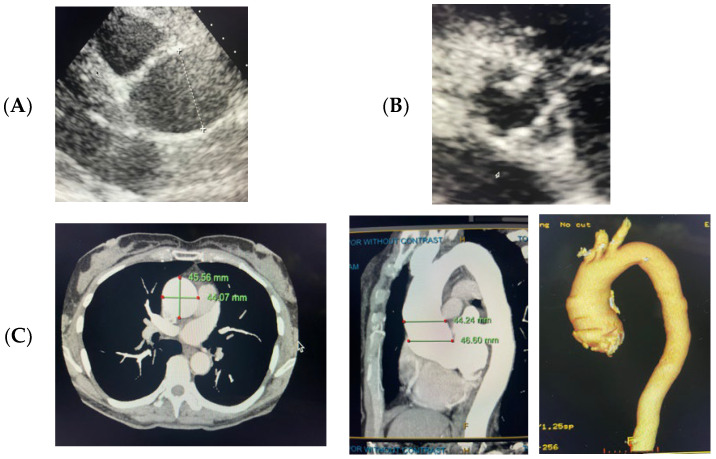
(**A**) Transthoracic echocardiographic PLAX view of aorta. (**B**) Transthoracic echocardiographic PSAX view of bicuspid aortic valve. (**C**) Aortic CTA with aortic measurements in 2D axial, 2D sagittal, and 3D reconstructions.

A 60-year-old Caucasian female with a history of a bicuspid aortic valve and ascending thoracic aortic aneurysm presented to our practice for a cardiac evaluation. Transthoracic echocardiogram (TTE) from an outside institution two years prior revealed an ascending aorta measuring 45 mm. At the time of our initial evaluation our patient was clinically asymptomatic and lived an active lifestyle without limitations. Initial physical examination was significant for a 2/6 graded holosystolic murmur over the right upper sternal border with radiation to the carotid arteries. To assess aortic valve function and the aortic anatomy, a TTE and computed tomography angiography (CTA) were performed. TTE revealed a bicuspid aortic valve with moderate calcific restriction of the superior leaflet as well as mild aortic valve insufficiency. Computed tomography angiography (CTA) revealed an ascending aorta measuring 46 × 45 mm with no other anomalies (Figure 1). We advised clinical genetic testing to exclude high-risk pathogenic mutations and to guide optimal clinical management.

### 2.2. Case 2

**Figure 2:**
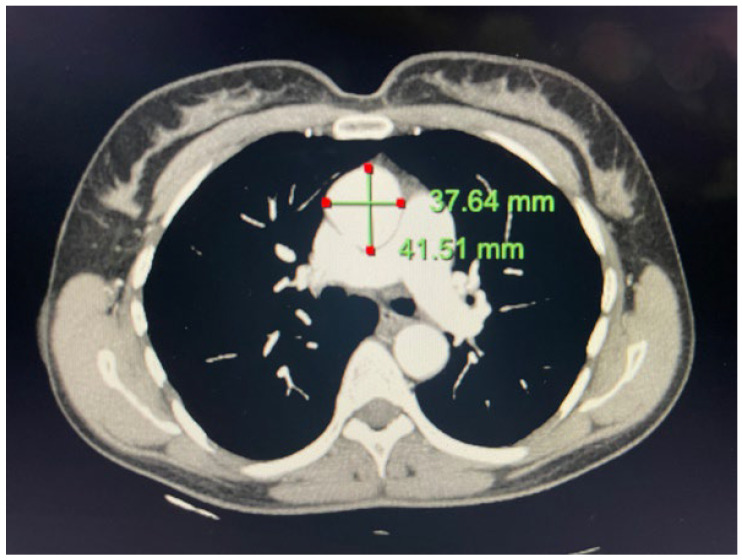
Aortic CTA with 2D axial slice demonstrating a 41.5 × 38 mm ascending aortic aneurysm.

A 62-year-old Caucasian female with no significant medical history was referred to our practice for evaluation following recent cardiovascular events in her family. A review of prior records was notable for an ascending aorta measuring 39 mm in 2020 on a EBCT and subsequently 40 mm on a CT scan performed in 2021 for a pulmonary evaluation. A transthoracic echo in our program revealed no anomalies of the aortic valve, with a mildly enlarged ascending aorta. We proceeded with a thoracic CTA, which measured her ascending aortic aneurysm at 41.5 mm, notable for a 2 mm increase in size of the aorta over three years (Figure 2).

The presenting TAA sizes of 4.6 cm and 4.15 cm with a mean of 4.35 cm +/− 0.25 cm found in our *PRDM5* patients were comparable to the size seen in patients with Marfan TAAs secondary to known pathogenic mutations in FBN1. In a sample of 78 patients with Marfan syndrome, notably due to mutations in the FBN1 gene, the average aneurysm diameter at presentation was 4.54 ± 1.3 cm, creating a presenting range of 2.2–8.3 cm [6]. 

## 3. Materials and Methods

Patients in our program with a history of aneurysmal or dissection diseases underwent clinical genetic testing (*n* = 145) to assess the mutation status of genes associated with vasculature, extracellular matrix, and aneurysmal/dissection disease to guide clinical management. Those with mutations in *PRDM5* (*n* = 2) are included as subjects in this study. Patients in this report underwent a clinical evaluation of thoracic aortic aneurysms and cardiac structure function using standard transthoracic echocardiography and thoracic CT angiographic techniques.

For genetic testing, we utilized a commercially available panel targeting 35 genes associated with thoracic aneurysmal and dissection diseases (TAAD) via genomic deoxyribonucleic acid-isolated salivary sampling [7,8,9,10,11,12,13]. This gene panel targets the detection of DNA sequence mutations in 35 genes (*ACTA2*, *BGN*, *CBS*, *CHST14*, *COL1A1*, *COL1A2*, *COL3A1*, *COL5A1*, *COL5A2*, *EFEMP2*, *FBN1*, *FBN2*, *FKBP14*, *FLNA*, *FOXE3*, *LOX*, *MAT2A*, *MED12*, *MFAP5*, *MYH11*, *MYLK*, *NOTCH1*, *PLOD1*, *PRDM5*, *PRKG1*, *SKI*, *SLC2A10*, *SMAD3*, *SMAD4*, *TGFB2*, *TGFB3*, *TGFBR1*, *TGFBR2*, *TNXB* (excluding exons 32–44), and *ZNF469*) via either Next-Generation or Sanger sequencing of all coding domains and well into the flanking 5′ and 3′ ends of all the introns and untranslated regions [7,8,9,10,11,12,13]. Gross deletion/duplication analysis can determine the gene copy number for the covered exons and untranslated regions of all genes (excluding *CBS* and *TNXB* exons 32–44) [7,8,9,10,11,12,13]. Bait-capture methods were utilized for the enrichment of coding exon sequences of interest using biotinylated oligonucleotide probes and subsequent polymerase chain reaction and sequencing, utilizing NCBI reference sequences [7,8,9,10,11,12,13]. Additional Sanger sequencing was performed for any regions missing or with any insufficient read depth coverage for reliable heterozygous variant detection. Variants in regions complicated by pseudogene interference, variant calls not satisfying the depth of coverage and variant allele frequency quality thresholds, and potentially homozygous variants were verified using Sanger sequencing. Gross deletion/duplication analyses were performed for all genes using a custom pipeline based on read-depth from NGS data, followed by a confirmatory orthogonal method, as needed [7,8,9,10,11,12,13]. Sequence analysis was based on the following NCBI reference sequences: *ACTA2* NM_001613.2, *BGN* NM_001711.4, *CBS* NM_000071.2, *CHST14* NM_130468.3, *COL1A1* NM_000088.3, *COL1A2* NM_000089.3, *COL3A1* NM_000090.3, *COL5A1* NM_000093.4, *COL5A2* NM_ 000393.3, *EFEMP2* NM_016938.4, *FBN1* NM_000138.4, *FBN2* NM_001999.3, *FKBP14* NM_017946.2, *FLNA* NM_001456.3, *FOXE3* NM_012186.2, *LOX* NM_002317.5, *MAT2A* NM_005911.5, *MED12* NM_005120.2, *MFAP5* NM_003480.2, *MYH11* NM_002474.2, *MYLK* NM_053025.3, *NOTCH1* NM_017617.3, *PLOD1* NM_000302.3, *PRDM5* NM_018699.2, *PRKG1* NM_006258.3, *SKI* NM_003036.3, *SLC2A10* NM_030777.3, *SMAD3* NM_005902.3, *SMAD4* NM_005359.5, *TGFB2* NM_003238.3, *TGFB3* NM_003239.2, *TGFBR1* NM_004612.2, *TGFBR2* NM_003242.5, *TNXB* NM_019105.6, *ZNF469* NM_001127464.1 [7,8,9,10,11,12,13]. Genetic counseling was provided on a 1:1 basis with each patient.

## 4. Genetic Results

### 4.1. Case 1 

We identified a variant of unknown significance (VUS), p.R83H variant (also known as c.248G>A), located on coding exon 3 of the PR/SET domain 5 (PRDM5) protein coding gene. A search of the gnomAD database did not reveal this VUS, reducing the likelihood that this is a cold variant or common finding [14]. This single nucleotide variant (SNV) resulted in an arginine-to-histidine replacement at codon 83, two amino acids with similar properties (Table 1). The Grantham Score, which helps predict the effect of substitutions between amino acids, was measured at 29. This low score suggests less evolutionary distance. This mutation provides potential for the disruption of extracellular matrix components through the disruption of the secondary structure, hydrophilicity, and polarity of PRDM5.

### 4.2. Case 2 

We identified a p.E129A variant (also known as c.386A>C) located on coding exon 4 of the PRDM5 gene. A search of the gnomAD database did not reveal this VUS, reducing the likelihood that this is a cold variant or common finding [14]. This SNV resulted in a change from glutamic acid to alanine at codon 129, two amino acids with differing properties (Table 2). The Grantham Score was moderately radical, at 107. Though this variant was predicted to be tolerated by in silico analysis, this variation provided potential for the structural disruption of extracellular matrix components through changes in the PRDM5 secondary structure, hydrophilicity, and polarity.

Following the sequencing of all 35 genes in the TAAD panel, PRDM5 was the only gene demonstrating a genetic variation in these cases, making this report the first to associate the clinical phenotype of aortopathy and single nucleotide variants in PRDM5. We cannot attribute causation, as this requires further detailed characterizations of these SNV regarding protein structure, resultant biochemistry, and demonstration of the tissue level effect. However, we anticipate that our work will generate interest in these mechanistic studies to demonstrate causal effects between genetic variants in PRDM5 on tissue changes within the extra cellular matrix that subsequently lead to aortopathy.

## 5. Discussion: PRDM5

The identification of genetic variants that are responsible for the hereditary development of TAAs is necessary for establishing molecular mechanisms that are responsible for aneurysm development [16]. Two major categories of gene alterations have been established, including mutations in transforming growth factor beta (TGF-β) signaling cascade components, also known as TGF-β vasculopathies. The second set of genetic mutations includes various components of the smooth muscle contractile apparatus [3]. Approximately 30 genes involved in the development of syndromic or non-syndromic TAA development have been established, with many causative mutations remaining unidentified [16].

**Figure 3:**
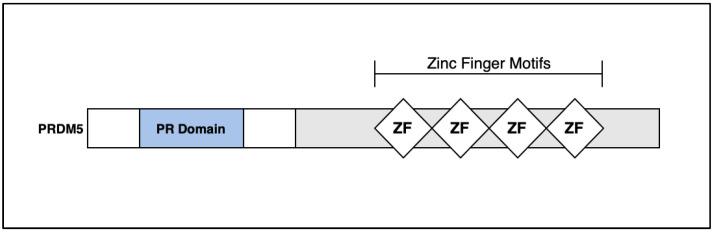
Simplified structure of the *PRDM5* gene. The gene, located on chromosome 4q27, is roughly 100 amino acids and contains 23 exons. Notable characteristics include a PR domain and multiple zinc finger motifs. PR-domain-containing proteins are frequently involved in transcriptional regulation.

The PR domain is a protein–protein interaction of roughly 100 amino acids, and PR-domain-containing proteins are often involved in transcriptional regulation [17]. Several PR-domain-containing proteins have been linked with cell differentiation and tumorigenesis [18]. The *PRDM5* gene is located on chromosome 4q27 and is a 23-exon protein coding gene. The protein encoded by the *PRDM5* gene is a transcription factor of the PR-domain protein family, and contains a PR-domain as well as multiple zinc finger motifs (Figure 3) [19].

**Figure 4:**
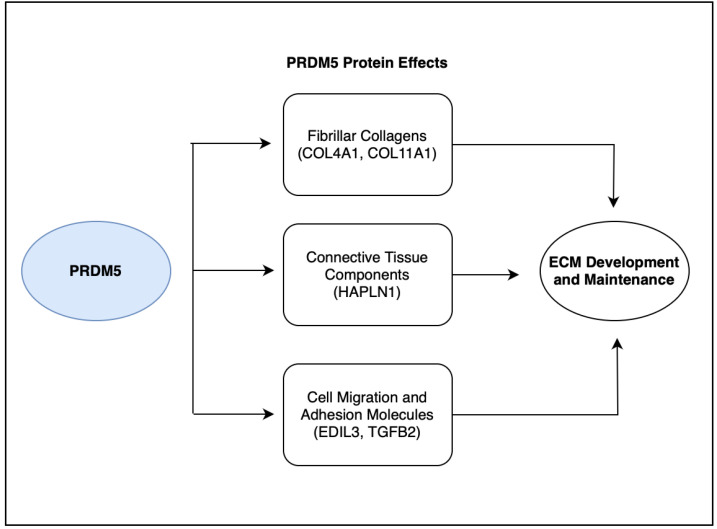
The *PRDM5* protein is responsible for ECM development and maintenance through downstream effects on various proteins and molecules. *PRDM5* plays a role in the regulation of fibrillar collagens such as *COL4A1* and *COL11A1*, connective tissue components including *HAPLN1*, and cell migration and adhesion molecules such as *EDIL3* and *TGFB2*. Downregulation of these downstream proteins and molecules in patients with *PRDM5* mutations is potentially responsible for ECM dysregulation and subsequent aneurysm formation.

The *PRDM5* protein was found to have a variety of functions as a transcription factor, being involved as a tumor suppressor gene in epithelial cancer and as a regulator of extracellular matrix development in corneal and bone cells [20]. The *PRDM5* transcription factor is critical for extracellular matrix (ECM) development and maintenance in the cornea. The downregulation of genes encoding fibrillar collagens (e.g., *COL4A1* and *COL11A1*), connective tissue components (e.g., *HAPLN1*), and molecules that regulate cellular migratory patterns and adhesion (e.g., *EDIL3* and *TGFB2*) was found in patients with *PRDM5* mutations on a microarray analysis of dermal fibroblasts. *PRDM5* mutations resulted in significant corneal fragility, and the central corneal thickness was markedly decreased [21]. We hypothesize that if a genetic variation in *PRDM5* encodes for an aberrant protein, a subsequent disruption in these protein functions (Figure 4) will lead to aneurysm development. Our laboratory has begun further exploration to explore these underlying mechanisms.

**Figure 5:**
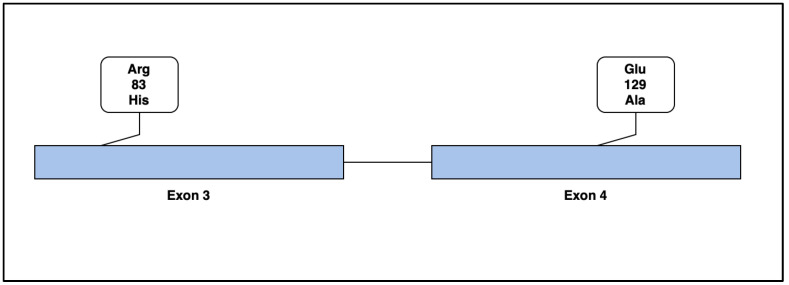
Mutations in protein-coding Exon 3 and Exon 4 of *PRDM5* were present in our subjects. An arginine-to-histidine substitution at codon 83 on Exon 3 was seen in Case 1, and a glutamic-acid-to-alanine substitution at codon 129 on Exon 4 was present in Case 2. We hypothesize that these substitutions led to a loss of function of the *PRDM5* protein, which affects ECM development and maintenance in our subjects, subsequently leading to aneurysm development.

*PRDM5* mutations in human beings are associated with the development of brittle cornea syndrome (BCS). BCS is an autosomal recessive disorder of the connective tissue that is characterized by both ocular and extra-ocular features. Notable phenotypic features including blue sclera, keratoconus, deafness, and joint hypermobility are present, with the suggested mechanism being a result of PRDM5′s role in extracellular matrix homeostasis. Further strengthening this linkage are the causal relationship between BCS and genetic mutations in another ECM regulating protein, ZNF469 [21,22], and our group’s recent reports associating variants in this gene with aortic and arterial aneurysmal and dissection disorders [23,24]. The disruption of the ECM via mutations in Exon 3 and Exon 4 of *PRDM5* supports our hypothesis that these variations may contribute to a loss of vascular integrity, leading to subsequent aneurysm formation (Figure 5).

Limitations of our study include the recognition that although the variants described in our report are missense mutations, we suspect that they are likely to encode for truncated proteins. Further, though it is unknown whether the variants described are in the cis or trans position, our expectation with these phenotypes is the trans position, thereby more likely coding for proteins with aberrant structure. The variants identified in the report were not present in gnomAD, and this significantly reduces the possibility of these being “cold” variants. We believe that further detailed mechanistic study will help to better define the role of the mutations we have discovered in subsequent protein biochemical function and the resultant phenotypic effects.

## 6. Conclusions

TAAs are clinically silent pathologies with potentially fatal complications, and an increasing amount of novel pathologic mutations are being discovered with further research. This report of two subjects is the first to make an association between *PRDM5* and TAA development and contributes to the evolving understanding of how mutations affecting extracellular-matrix-encoding genes may lead to vascular aneurysms. Further research establishing the pathogenicity of *PDRM5* mutations in aneurysm formation will advance screening and care for patients with this disease state.

## Figures and Tables

**Table 1:** Comparison of amino acid properties present in p.R83H substitution [15].

Case 1 Amino Acid Characteristics	Arg (R)	His (H)
Amino Acid Name	Arginine	Histidine
Polarity/Charge	Positive Charge	Positive Charge
pH	Basic	Basic
Residue Weight	156	137
Hydrophobicity Score	−4.5	−3.2
Hydrophilicity Score	3	−0.5
Secondary Structure Propensity	α indifferent/β indifferent	Weak α former/β former

**Table 2:** Comparison of amino acid properties present in p.E129A substitution [15].

Case 2 Amino Acid Characteristics	Glu (E)	Ala (A)
Amino Acid Name	Glutamic Acid	Alanine
Polarity/Charge	Negative Charge	Non-Polar
pH	Acidic	Neutral
Residue Weight	129	71
Hydrophobicity Score	−3.5	1.8
Hydrophilicity Score	3	−0.5
Secondary Structure Propensity	Strong α former/strong β breaker	Strong α former/β indifferent

## Data Availability

The data presented in this study are available on request from the corresponding author. The data are not publicly available due to patient privacy considerations.

## References

[1] Senser E.M., Misra S., Henkin S. (2021). Thoracic Aortic Aneurysm: A Clinical Review. Cardiol. Clin..

[2] Martin-Blazquez A., Heredero A., Aldamiz-Echevarria G., Martin-Lorenzo M., Alvarez-Llamas G. (2021). Non-syndromic thoracic aortic aneurysm: Cellular and molecular insights. J. Pathol..

[3] Isselbacher E.M., Lino Cardenas C.L., Lindsay M.E. (2016). Hereditary Influence in Thoracic Aortic Aneurysm and Dissection. Circulation.

[4] Salameh M.J., Black I.J.H., Ratchford E.V. (2018). Thoracic aortic aneurysm. Vasc. Med..

[5] Pinard A., Jones G.T., Milewicz D.M. (2019). Genetics of Thoracic and Abdominal Aortic Diseases. Circ. Res..

[6] Saeyeldin A., Zafar M.A., Velasquez C.A., Ip K., Gryaznov A., Brownstein A.J., Li Y., Rizzo J.A., Erben Y., Ziganshin B.A. (2017). Natural history of aortic root aneurysms in Marfan syndrome. Ann. Cardiothorac. Surg..

[7] Jarvis M. (2006). TAADNext: Analyses of 35 Genes Associated with Thoracic Aortic Aneurysms and Dissections.

[8] Antolik C. (2021). TAADNext: Analyses of 35 Genes Associated with Thoracic Aortic Aneurysms and Dissections.

[9] Geng G. (2022). TAADNext: Analyses of 35 Genes Associated with Thoracic Aortic Aneurysms and Dissections.

[10] Kou E. (2020). TAADNext: Analyses of 35 Genes Associated with Thoracic Aortic Aneurysms and Dissections.

[11] Antolik C. (2022). TAADNext: Analyses of 35 Genes Associated with Thoracic Aortic Aneurysms and Dissections.

[12] Geng J. (2020). TAADNext: Analyses of 35 Genes Associated with Thoracic Aortic Aneurysms and Dissections.

[13] Antolik C. (2020). Gene Sequence & Deletion/Duplication Analyses of FBN1 Reflex to TAADNext.

[14] gnomAD. https://gnomad.broadinstitute.org/gene/ENSG00000138738?dataset=gnomad_r2_1.

[15] TAAD Syndrome Genetic Testing | TAADNext | Ambry Genetics. https://www.ambrygen.com/providers/genetic-testing/12/cardiology/taadnext.

[16] Creamer T.J., Bramel E.E., MacFarlane E.G. (2021). Insights on the Pathogenesis of Aneurysm through the Study of Hereditary Aortopathies. Genes.

[17] (2000). The yin-yang of PR-domain family genes in tumorigenesis. Histol. Histopathol..

[18] PRDM5 PR/SET Domain 5 [Homo Sapiens (Human)]—Gene—NCBI. https://www.ncbi.nlm.nih.gov/gene/11107.

[19] PRDM5 Gene—GeneCards|PRDM5 Protein|PRDM5 Antibody. https://www.genecards.org/cgi-bin/carddisp.pl?gene=PRDM5.

[20] Dhooge T., Van Damme T., Syx D., Mosquera L.M., Nampoothiri S., Radhakrishnan A., Simsek-Kiper P.O., Utine G.E., Bonduelle M., Migeotte I. (2021). More than meets the eye: Expanding and reviewing the clinical and mutational spectrum of brittle cornea syndrome. Hum. Mutat..

[21] Wright E.M.B., Spencer H.L., Daly S.B., Manson F.D., Zeef L.A., Urquhart J., Zoppi N., Bonshek R., Tosounidis I., Mohan M. (2011). Mutations in PRDM5 in Brittle Cornea Syndrome Identify a Pathway Regulating Extracellular Matrix Development and Maintenance. Am. J. Hum. Genet..

[22] Porter L.F., Gallego-Pinazo R., Keeling C.L., Kamieniorz M., Zoppi N., Colombi M., Giunta C., Bonshek R., Manson F.D., Black G.C. (2015). Bruch’s membrane abnormalities in PRDM5-related brittle cornea syndrome. Orphanet J. Rare Dis..

[23] Wolf A., Khimani F., Sathyamoorthy M. (2022). Identification of Single-Nucleotide Polymorphisms in ZNF469 in a Patient with Aortoiliac Aneurysmal Disease. Cardiogenetics.

[24] Wolf A., Hong C., Sathyamoorthy M. (2023). The Novel Phenotype-to-Genotype Association of Novel Single-Nucleotide Variants in Exon 1 and Exon 2 of the Collagen Matrix-Encoding Gene ZNF469 to Arterial Aneurysmal and Dissection Diseases.

